# Birth preparedness and complication readiness – a qualitative study among community members in rural Tanzania

**DOI:** 10.3402/gha.v8.26922

**Published:** 2015-06-12

**Authors:** Furaha August, Andrea B. Pembe, Edmund Kayombo, Columba Mbekenga, Pia Axemo, Elisabeth Darj

**Affiliations:** 1 Department of Obstetrics and Gynaecology, Muhimbili University of Health and Allied Sciences, Dar es Salaam, Tanzania; 2 Department of Women's and Children's Health, International Maternal and Child Health, Uppsala University, Uppsala, Sweden; 3 Institute of Traditional Medicine, Muhimbili University of Health and Allied Sciences, Dar es Salaam, Tanzania; 4 Department of Community Health Nursing, School of Nursing, Muhimbili University of Health and Allied Sciences, Dar es Salaam, Tanzania; 5 Department of Public Health and General Practice, Norwegian University of Science and Technology, Trondheim, Norway

**Keywords:** perceptions, birth preparedness and complication readiness, rural Tanzania

## Abstract

**Background:**

Birth preparedness and complication readiness (BP/CR) strategies are aimed at reducing delays in seeking, reaching, and receiving care. Counselling on birth preparedness is provided during antenatal care visits. However, it is not clear why birth preparedness messages do not translate to utilisation of facility delivery. This study explores the perceptions, experiences, and challenges the community faces on BP/CR.

**Design:**

A qualitative study design using Focused Group Discussions was conducted. Twelve focus group discussions were held with four separate groups: young men and women and older men and women in a rural community in Tanzania. Qualitative content analysis was used to analyse the data.

**Results:**

The community members expressed a perceived need to prepare for childbirth. They were aware of the importance to attend the antenatal clinics, relied on family support for practical and financial preparations such as saving money for costs related to delivery, moving closer to the nearest hospital, and also to use traditional herbs, in favour of a positive outcome. Community recognised that pregnancy and childbirth complications are preferably treated at hospital. Facility delivery was preferred; however, certain factors including stigma on unmarried women and transportation were identified as hindering birth preparedness and hence utilisation of skilled care. Challenges were related to the consequences of poverty, though the maternal health care should be free, they perceived difficulties due to informal user fees.

**Conclusions:**

This study revealed community perceptions that were in favour of using skilled care in BP/CR. However, issues related to inability to prepare in advance hinder the realisation of the intention to use skilled care. It is important to innovate how the community reinforces BP/CR, such as using insurance schemes, using community health funds, and providing information on other birth preparedness messages via community health workers.

The World Health Organisation (WHO) has a vision of universal coverage of health care; however, due to inequities in functioning health systems, pregnant women in the developing world have a high risk of morbidity and mortality (1). The adult lifetime risk of maternal mortality in women in the sub-Saharan region is estimated to be 1 in 38 (2). This figure is in sharp contrast to that of the developed world, which stands at 1 in 3,700. Additionally, the maternal mortality ratio (MMR) in sub-Saharan countries is high, at 510 per 100,000 live births, contributing to 62% of maternal deaths in the world (2). Although progress has been made in the reduction in infant mortality and neonatal mortality, challenges still remain with the reduction of maternal mortality (3). It is estimated that only 16 countries will achieve the target of reducing maternal mortality by 75% by 2015 as per the Millennium Development Goal (MDG-5) (4). In spite of these discouraging figures, there are proven interventions that are known to prevent maternal deaths. These include skilled attendance for pregnancy and childbirth, access to emergency obstetric care, and the use of family planning (5–7).

Tanzania, as a sub-Saharan county, contributes to the high number of maternal deaths and has a high MMR, although it has been reduced in the most recent estimate to 454 per 100,000 live births from 578 per 100,000 in 2005 (8). The government has been active in formulating policies and strategies to address this burden (9), including the introduction of focused antenatal care (FANC) (10) in 2002 as recommended by the WHO. Individual counselling of pregnant women on Birth Preparedness and Complication Readiness (BP/CR) is one of the components of FANC. BP/CR comprises principles that encourage women and families to make decisions before the onset of labour and in case of obstetric complications. Knowledge of danger signs, identifying a mode of transport, saving money, identifying a skilled attendant, identifying where to go in case of complications, and identifying a blood donor are all elements of BP/CR (11, 12).

Whilst it is assumed that knowledge of obstetric danger signs and birth preparedness address the delays in seeking care and reaching care (13), studies completed in developing countries have shown that women have poor knowledge of danger signs (14–18). Moreover, other studies in sub-Saharan countries have also found low knowledge of birth preparedness and the practice of BP/CR (19–21). This raises the question of whether women are really counselled on danger signs and the meaning of birth preparedness during their antenatal care (ANC) visits. In Tanzania, it has been demonstrated that few women attending ANC are counselled on obstetric danger signs during their very short visits (22, 23).

Furthermore, counselling on BP/CR during ANC is envisaged to increase facility delivery, as was demonstrated in studies from Bangladesh and Burkina Faso (24–26). A recent cluster randomised trial on the introduction of a birth plan in northern Tanzania demonstrated an increase in facility deliveries in the intervention area as compared to the control area (27). However, in contrast, other studies, including a systematic review of the effect of BP/CR interventions, have shown no or modest effect on facility delivery (28, 29). The reason for these findings is not clear. The aim was to explore the perceptions, experiences, and challenges faced by the community regarding BP/CR.

## Theoretical framework

To understand the needs for seeking health care, several theoretical framings have been used including the Health Belief Model (HBM), the theory of reasoned action, and the theory of planned behaviour. The theory of planned behaviour developed by Fishbein and Azjen (30) posits that performing a behaviour is proximally determined by the intention to perform the behaviour. The HBM, developed by US psychologists in the 1950s Hochbaum, Rosenstock and Kegel and modified to include self-efficacy (31), has been used by researchers since then, to structure, explain, and predict the health behaviour of individuals when faced with a health problem that is perceived as threatening (32). The HBM explores the health-related actions a person will take when someone perceives a negative and severe health condition such as, a woman can die due to obstetric complications, and, realising the benefits if it is avoided, and following recommended actions. Cues for actions that would activate readiness and self-efficacy involve supporting the confidence of the individual to act accordingly. We will frame our discussions using this model.

## Materials and methods

### Study design

A qualitative study design using focus group discussions (FGDs) was employed (33) to explore the perception of the community on BP/CR.

### Study setting

This study was carried out in the Rufiji district located in the eastern part of Tanzania. The 2012, population census estimates that the district has about 217,000 inhabitants (34). The main source of livelihood is subsistence agriculture of cassava and maize. There is also small-scale fishing in the flood plains part of the district. The district has road networks but during the rainy season, they are impassable and hence inaccessible by car. The main mode of transportation is bicycles and motorcycles. Public transport is available along the main road to Dar es Salaam, the commercial centre of Tanzania. Women usually walk to ANC clinic. Motorcycles are utilised to reach health facilities during labour and in case of an obstetric emergency.

The primary frontline health facilities in the district are dispensaries and health centres. In total, there are 52 dispensaries and four health centres. Two hospitals serve as referral facilities in this district; one is government-owned and the other a private not-for-profit hospital owned by a mission. Both hospitals can provide medical and surgical care, including comprehensive emergency obstetric care, such as caesarean section and blood transfusion. ANC and delivery services are provided in hospitals, all health centres and dispensaries. During the ANC visits, the women are supposed to be given health education on BP/CR individually.

### Participants

Participants were purposefully selected and invited from the community. Homogenous groups were selected and included older women and men, and young women and men, to allow free discussion among the groups (35). Separating older and young people into different groups helped to remove the shyness that young people will have of speaking in front of their elders, as well as to minimise the feelings of insubordination. The village chairpersons in these communities helped to identify the participants. The participants represented individuals from both semi-urban and rural areas. Young women and men of less than 40 years of age were included if they had had a baby delivered within the last 2 years. Older men and women, who were grandfathers and grandmothers, were recruited if they were more than 50 years old. After initially conducting FGDs among each group of young and older men and women in two separate villages, we included an extra village with further FGDs to ensure that saturation was reached. In total, 65 young women and men, as well as 67 older men and women, participated in the FGDs (Table 1).

**Table 1:** Participants’ characteristics

Participants	Age range	Average age	Number of participants	Number of FGDs
Young men	20–36	23	33	3
Young women	19–32	24	32	3
Older men	51–76	55	32	3
Older women	53–74	52	35	3

### Data collection

Conducting FGDs was chosen as the data collection method to capture perceptions within the community as they involve group interactions and can give rich information about perceptions and experiences related to BP/CR, which have relevance to the group in a way that a questionnaire would not have been able to capture. Twelve FGDs were conducted during July and August 2011. The FGDs had 10–12 participants in each group. There were three FGDs for each group of older men and women and young men and women. A pretested topic guide was used to conduct the FGDs. The topics for discussion included cultural and social issues surrounding birth preparedness, pregnancy, and childbirth, steps taken to prepare for pregnancy and potential complications during childbirth in the community, recognition of maternal danger signs, and the use of traditional medicine during pregnancy and childbirth.

The FGDs were conducted in Swahili and were audio-recorded and lasted between 1 and 1.5 h. All FGDs were led by a moderator (EK or FA) and notes were taken by an observer (ABP or FA). EK is an anthropologist with experience in qualitative research and maternal health. FA is an obstetrician/gynaecologist and lecturer with experience in reproductive health. ABP is an obstetrician/gynaecologist, senior lecturer and researcher, and has previously conducted studies on maternal referrals in this area. The FGDs were conducted in the dispensary/village office or under a cashew nut tree, where privacy was ensured. The moderator would start the discussion by asking the participants to talk about what happens when one realises that a woman in their family is pregnant. How do they proceed with starting preparations for childbirth and how might they prepare for any emergency relating to pregnancy and childbirth?

### Data analysis

Prior to analysing the data, all FGDs were transcribed verbatim in Swahili and then translated into English by the first author and a research assistant, so that the members of the research team who do not speak Swahili could contribute to the analysis of the material. To ensure fidelity, an independent translator translated two of the FGDs back into Swahili.

A qualitative content analysis approach, as described by Graneheim and Lundman, was used to analyse the data (36). The transcripts were transferred into the NVivo 10 software package (QSR International) to aid in the sorting of the data. The focus of the analysis was to obtain the manifest meaning of the material. The FGDs text was read several times to allow the research team to become familiar with and understand the material. Meaning units were identified in relation to the aim of the study. The meaning units that were extracted from each topic of the analysis were then condensed and from these condensed meaning units, codes and subcategories were developed and then combined together to form categories depending on their differences and similarities. All authors read the material and reflected on each of the meaning units, condensed units, codes, subcategories, and categories until agreement was reached. An example of the analytic process is shown in Table 2.

**Table 2:** Example of the analytical process

Meaning units	Condensed meaning unit	Code	Subcategory	Category
‘Yes, there are dangerous symptoms. One is when an expecting mother gives out a lot of blood even before time of delivery. The best solution is to rush her to hospital where there are doctors who would advise you on what to do’	There are dangerous symptoms. The best solution is to rush her to hospital	Dangerous symptoms occur. Rush her to hospital	Recognising danger signs	Danger signs demand hospital care

### Ethical considerations and ethical clearance

Ethical approval to conduct the study was obtained from the Senate Publication and Research ethical committee of the Muhimbili University of Health and Allied Sciences (MUHAS). Permission was also granted from the District Executive Director of Rufiji District. The participants were told the aim of the study and that their participation was voluntary and they could withdraw at any time, without giving any reason. They were aware that although the conversation was audio-recorded, their identity would not be revealed. The information that they provided would only be used for research purposes and would not to be disclosed to anyone. Verbal consent to participate in the FGDs was given by each of the participants.

## Findings

During the analysis, two categories emerged from the FGDs, related to BP/CR: ‘All births need to be prepared’ and ‘Danger signs demand hospital care’, each divided into several subcategories. Quotes are given to illuminate the views of the participants (Table 3).

**Table 3:** Categories and subcategories on perception of birth preparedness and complication readiness

1. All births need to be prepared Antenatal care prioritised but has challenges Family responsible to support the pregnant mother Unmarried women stigmatised affecting preparation Practical preparations financially demanding Facility delivery preferred
2. Danger signs demand hospital care Recognising danger signs Health care services, ‘free but not free’ Men decide but in consultation Traditional beliefs are fading out Herbal remedies still used for treatment

## All births need to be prepared

### ANC is prioritised but has challenges

Participants reported that a woman first attends the nearest clinic providing ANC services for check-up when the term of the pregnancy is about 5 months. It was perceived to be important to attend the clinic to discover the stage of the pregnancy, to undergo some medical investigations, and to obtain some medications such as anti-malarial drugs suitable for use during pregnancy. Obtaining an antenatal card was important in case the woman requires further treatment in another hospital. It was also reported that they attend the clinic to receive professional advice on how to proceed with the pregnancy, especially if one might need to be referred to hospital for delivery.

When I was told that my daughter or granddaughter was pregnant, the first thing was to take her to hospital to check whether she really was pregnant. She would undergo a general health check-up. She would continue to attend clinic as her pregnancy grows. Her doctor would advise her on what to do, whether to go and deliver at a normal clinic or not. (Older woman FGD 4)

In the past, it was perceived that women who attended the clinic were only those with childbirth problems. First attendance towards the end of the pregnancy was the norm. Some participants revealed that they were born at home as their mothers did not attend clinic at all. But currently, most women do attend ANC clinic with only a few failing to attend.

In the old days, people had certain faiths. People did not bother checking their pregnancies then. Some of us were just born at home. Maybe they did not have many childbirth problems then. But now the situation is different. It's rare to find someone not going to check the progress of her pregnancy. (Young man FGD 3)

Reasons cited for not attending ANC include distance to the health facility and lack of money for transport, lack of nice clean clothes because the partner could not provide these, and advanced age of the pregnant woman. Fear of HIV testing for partners during the ANC was also mentioned. For the HIV test, the pregnant women are required to bring along their partners. Some health workers may at times refuse to provide ANC services to the women if they do not bring their partners.

As for others, they may even want to borrow clothes they wear … so her colleagues may laugh at her and joke with her telling her that ‘your husband was able to make you pregnant, how come he cannot afford to buy you clothes?’… (laughter). So, because of financial problems, others don't even have clothes to put on so that they would go to the clinic. (Young man FGD 7)

My partner refused to come with me. The nurses chased me away three times because I could not come with my partner. So I decided not to go, because my spouse refused, so why should I go there? (Young woman FGD 1)

### Family responsible to support pregnant mother

Supporting pregnant women was reported to be the responsibility of the family. The support could be in the form of helping the mother to perform household chores such as cooking, searching for firewood, and fetching water. The mother-in-law or aunts often perform these functions. Rest for the mother was described as being very important for the baby to grow well.

Another type of support reported by participants is that provided during labour. The mother-in-law, aunt, or sisters-in-law are readily available to escort pregnant women to the hospital for delivery when labour starts. This was described as being important because if the delivery occurs on the way to hospital, the elder women would be able to assist in the delivery of the baby.

That's why we said love has to be there in the family … so, anyone who is there … be it your sister-in-law, your mother may be there but she is already frail, so even your sister-in-law will be there; even a neighbor, provided that you trust her. She may be able to help you, escort you and if God wishes, you get there fine, even if you deliver on the way. (Older woman FGD 8)

### Unmarried women stigmatised affecting preparation

Mothers of unmarried women described feeling responsible for helping by making sure their daughters attend clinic and eventually have a safe delivery. Other participants were critical that the young girls nowadays do not listen to what they are told by their parents in terms of engaging in sex and becoming pregnant before marriage. As a result, the girls do not disclose their pregnancy to parents and may not attend ANC and deliver at home.

My daughter got pregnant and she did not tell us about that. On the day of delivery, she was alone in the room and delivered a male baby alone. We then saw the bed sheet that the child was covered with; we took that child and luckily for the Almighty God, that child is well to date. And till today, the daughter has not mentioned which man gave her the pregnancy. (Older woman FGD 8)

### Practical preparations financially demanding

When the pregnancy is confirmed, the partner will start to save money to prepare for the pregnancy. The participants discussed how the father or the mother of the partner would keep the money in a safe place. The men are seen as financial providers and are expected to work towards making money to ensure the supplies for the delivery are available. These include surgical gloves, threads for stitching, basins, surgical blades, cord ties, and clean clothes. Health workers routinely request that the pregnant mother ensures that they bring these supplies for the delivery. If men could not save money and provide this support, they were seen as irresponsible.

Once my wife tells me that she is pregnant; there are things that I start preparing for the baby's delivery. I buy gloves and razor blades. I buy clothes and ensure that my wife will deliver at a good place. (Young man FGD 6)

The money saved will also be used to hire transportation, although the participants reported that they do not always prepare in advance what transport they will use. Although they reasoned that motorcycles are not ideal, they did report using them to transport pregnant women in labour or in an emergency because no other affordable transport is available. The average cost for a trip by motorcycle is between 15,000 and 20,000 Tanzania shillings (9–12 USD).

The participants also reported that they were not aware of any organised community mechanism such as a community emergency fund at village level to help those who needed help in an emergency situation.

As part of birth preparedness, a lot of effort is taken by family members to have enough money to move the pregnant woman closer to the referral hospital. Usually, primiparas are advised to attend a referral hospital for delivery. Some participants reported that women are taken to relatives who live in Dar es Salaam, which is about 180 km from some of the areas in this study. Others discussed having to take the pregnant women nearer to the district hospital and to rent a room there, which can be expensive and challenging.

Our wives are still very young, so most of the time we are referred to a higher hospital – district hospital. And we are told about this quite early even before conditions become critical. So, when you note that she is almost due, you move close to the hospital and camp there … Some of us are of a poor economic condition, and at the district hospital, you may be required to rent a room or find a relative so they would take both you and your wife in until the time your wife delivers …. (Young man FGD 10)

### Facility delivery is preferred

It was discussed that it is essential to make preparations for delivery at the hospital. Hospital delivery was perceived to be safer than home delivery, and complications were seen to be dealt with professionally and quickly. Confidence in the competency of the well-trained health workers was also expressed. They also stated that, in this community, few women deliver at home. This is a different situation than in the past when the previous generation tended to deliver at home.

These days, we have health workers who are readily available. The government has trained a good number of midwives. And, they live with us in our streets; so, they can handle complications and refer the difficult ones. These people were appointed and then given the training. And most of the time they have all the necessary equipment, the plastic gloves and everything else. (Young man FGD 2)

Giving birth at home was perceived to be associated with some complications, such as severe pain, especially after delivery. In the hospital, women are given pain management injections and hence they do not suffer as much with the pain as compared to in the past when they would have suffered more as a result of delivering at home.

I once witnessed a woman after giving birth and she could not fall asleep. That happened because she gave birth at home, but when they give birth at a hospital … they are injected and suffer from no abdominal pains. In the old days, they used to suffer a lot and would cry out loudly. (Young man FGD 6)

Although facility delivery is preferred, at times the intention to do so was not always followed. Traditional birth attendants (TBAs) are consulted in the case of home deliveries. They live within the community and hence they are readily accessible and can come quickly to the home. They are able to assist the delivery and can recommend referral if they feel they cannot deal with a complicated delivery.

For instance when all the signs show that it's time, you go to the TBA and tell her that your wife is about to give birth and all the signs are there, she complains of pains and the like. So, she inspects her and finds that it's true that the woman is about to give birth. She may advise you of going to the hospital, but then the expecting mother is almost ready, so, the TBA may decide to help her right there … (Young man FGD 10)

A TBA who was participating in the discussions also alluded to this fact.

If it happens that the baby comes introducing buttocks first then you advise the father or grandfather of the girl, they discuss with one another that there is a difficult situation and we have to go with her to the dispensary in a hurry since I don't have that capability. (Older woman FGD 8)

Distance, quick onset of labour, and lack of money for buying supplies were given as reasons for women not using facility delivery. Failure to attend ANC was believed to disqualify the mothers from obtaining services for delivery at a health facility. In addition, some reported that they do not prepare transportation in advance because their economic situation does not allow them to.

Well, some live quite some distance from the health centre and at the same time they don't even have access to a bicycle … so she will deliver right there … she doesn't get to the health centre. This is the main obstacle. (Older woman FGD 4)

## Danger signs demand hospital care

### Recognising danger signs

The community members did mention some complications that may occur during pregnancy and these include excessive vaginal bleeding during pregnancy, fits during pregnancy, fever, rupture of membranes, leaking of urine, and prolonged labour. In the past, conditions such as fits were perceived to be associated with evil spirits; hence, these were treated with traditional medicine. The focus groups reported that these conditions are now treated in the hospital. Participants expressed that most of the conditions that they mentioned are better managed by professionals in the health facility.

Yes, there are dangerous symptoms. One is when an expecting mother gives out a lot of blood even before time of delivery. The best solution is to rush her to hospital where there are doctors who would advise you on what to do. (Older woman FGD 4)

### Healthcare services, ‘Free but not free’

Despite the fact that the community prefers to go to a health facility, they reported that they are not happy when they attend the clinic as they have to pay for consultations and then they are told to buy drugs. It is known by the community that pregnant women should receive free services. They are suspicious that the pharmacies where they are sent to buy delivery equipment and drugs belong to the same health workers. Furthermore, they feared that the health worker might refuse to treat the woman during delivery, until she provides the necessary supplies, such as gloves. Because of their poor economic status, some cannot afford to bring the supplies. This provided a dilemma for the participants who then asked why they bothered to come to the health facility at all.

You went there so that she would help you, instead she sends you to go and buy gloves so she would make some tests on you. The situation you left at home was not good financially, yet she wants you to go home first and ask your husband to go and buy gloves for you then go back to the health centre. At the same time, your time to give birth is almost up and you're in a critical condition. So, sometimes you think it would be better for you to remain seated where you are so that you would give birth right there alone … unless a neighbour passes by and is kind enough to help you … (Young woman FGD 1)

In some other situations, the doctors were reported to randomly ask for unexplained payment to provide quick services in case of delivery. If the money was not provided, the woman might not receive the required service. Participants were also unhappy that the health staff retained the unused supplies that they brought to hospital for delivery. The money that was used to buy these extra supplies could have been used for buying other things such as food.

When you go the doctor and ask him to look at your wife … that's when he will tell you what to bring, kerosene oil, soap and several others … that's when he will start working on your wife … but if you go empty handed … no service. So, it's a form of corruption. After the service, he will tell you, ‘See? I've taken care of your wife, where is my “asante”?’(Swahili word literary means where is my ‘thank you’) So, you give a bribe when going in and also when going out. (Older man FGD 7)

I use one or two pairs for investigating and for delivery, you see? Now those that remain should be returned to you, but they don't return them. Where do they go? I could have used the money to buy food. (Young woman FGD 5)

Furthermore, in this community, preparation for identifying a suitable blood donor is not done in advance. In case of an emergency, when a blood transfusion is needed, usually the family looks for a relative who has a matching blood group if an emergency arises. If one is not found, then they talk to the health workers at the facility. The participants reported that whenever they do not secure a blood donor, they might get it from the health workers. They must then pay for the required blood transfusion at the facility.

Also, before donating blood … donors have to be checked on everything … to ensure that the blood is safe so that the right blood groups would be found. So, we will pay money for blood only when all of the people who wanted to donate their blood, happened not to be of the right blood group. (Older man FGD 11)

### Men decide but in consultation

Participants reported that the decision to go to health facility for delivery or in case of an emergency is usually made by the husband/partner but in consultation with other members of the family like the grandmother or mother-in-law. In this community, the pregnant mother will go to live with her parents or parents-in-law when approaching delivery time. When the woman is in labour or in an emergency, the pregnant woman would alert her mother or mother-in-law. This elderly woman would then discuss the situation with the husband or the father-in-law and decide whether and how to go to the hospital. If he is not around, others make the decision on behalf of him.

The one who decides to go to the hospital is your husband. However, it is normal for the family to discuss and come with a decision. (Young woman FGD 5)

The first that counts is your husband. However, it's normal that the family will discuss about your condition when you happen to face problems. They'll sit down and come up with the best decision, that is, regarding this problem … let's do this and that and that. (Young woman FGD 5)

### Traditional beliefs are fading away

The community also explained that in the past they used to believe that eating certain foods such as eggs was detrimental to the unborn foetus. But nowadays, those beliefs are no longer entertained. For birth preparedness, women can basically eat anything that is available. However, there was a belief that if a woman has had repeated abortions, then this is due to evil spirits and the woman must see a traditional healer for the treatment. The herbs prescribed by the healer are believed to help to ‘tie’ the pregnancy.

Maybe for just some people, but most of us don't have. There are those who truly have problems, each time they become pregnant, the pregnancies are aborted. Such a person may go to a traditional healer so that she would be ‘tied’. (Young woman FGD 1)

### Herbal remedies are still used for treatment

Participants reported that they use traditional herbs in some situations, such as during labour. These are used especially when delivery takes place at home. It is believed that the herbs prevent prolonged labour. Elders in the family have the knowledge on how to prepare the herbs when the woman goes into labour. Even at the hospital, this medication can be given if the woman is seen to have a prolonged labour.

If she is taken to the hospital at 1 am she remains there until the next day at around 9 am and she would not yet have given birth … and still complains of the deep pains, we call it ‘Ngwega’. So, in order to avoid all these, the medicine is boiled, she is made to drink the juice and after about 3 h, everything goes fine. So, she gives birth safely. (Young woman FGD 1)

## Discussion

Our study has found that on BP/CR, the community perceived the importance of attending ANC, family is responsible in helping the pregnant woman, financial and practical preparations are essential, and all these should preferably lead to facility delivery. Additionally, community identified danger signs that are best managed at the hospital, though there are challenges such as healthcare services not being really free and the use of traditional herbs during pregnancy and childbirth or emergency. We frame our discussion using the HBM within the following dimensions: perceived severity and susceptibility, perceived benefits, perceived barriers, cues for action, and self-efficacy.

### Perceived susceptibility and perceived severity

Obstetric complications such as excessive haemorrhage, fits during pregnancy, and malaria were mentioned as being serious enough to require immediate care. Therefore, participants have shown that there is a perceived susceptibility and severity that refers to individual or community perceptions of risks of maternal or perinatal death in the incidences of not accessing skilled care. The community members understood that these conditions could affect anyone and may lead to death. However, financial power and gender roles contribute to inadequate birth preparedness (22, 37–39). A previous study in the same region showed that men are the decision-makers concerning complying with referral advice, and the woman have little or no voice in these decisions, regardless of her becoming suspicious of a potentially dangerous situation during pregnancy or delivery (40). In our study, we also found that men are decision-makers. Nevertheless, it is encouraging to find that couples discussed these perceived risks, which made them prefer to seek facility delivery and learn how to prepare for childbirth, as has been shown in other studies from low-income countries (41, 42).

### Perceived benefit

Perceived benefit is related to the belief that taking action will reduce the risk. It is known that ANC provision improves maternal and neonatal outcomes (43). We have found that women and their families expressed a preference to attend the health facility for ANC services and for delivery. The perceived benefits of attending ANC were having knowledge of whether they were truly pregnant, medical tests, medication for malaria prevention, getting professional advice on where to deliver, and obtaining their antenatal card. Similar findings have been observed in other low-income countries (39, 44–47). This finding implies that there is an awareness within the community that there is a benefit to using the ANC services and seeking skilled birth attendants at health facilities during labour to avoid fatal complications related to childbirth, as has been demonstrated with recent studies from other African settings, such as Malawi and Ethiopia (46, 48). The competency of the health workers and effective management of potential complications such as pain after delivery were some of the essential perceived benefits in facility delivery, as mentioned in other studies (49–51).

### Perceived barriers

Perceived barriers are those that are identified to impede the use of skilled attendance. The barriers to utilisation of ANC services that have been observed, such as financial challenges and distance to health facility, are in agreement with other studies (46, 47, 52, 53). In addition, our study has found that pregnant women do not attend ANC due to various reasons such as lack of good clothes, which is attributed to the failure of the husband to buy these for his wife. This perception may be explained by the fact that there is increased exposure to multimedia platforms that encourage aesthetic appearance, even to attend clinic, financial difficulties, or the influence of modernity such as the expectation to wear trendy clothes as reported by Wight in northern Tanzania (54).

Interestingly, counselling and testing for HIV were also found to be a possible deterrent to attend ANC, because of some men's refusal to attend ANC. In Tanzania, men are encouraged to attend ANC together with their partners for counselling and testing for HIV in pregnancy. Failure to bring a partner may invite rebuke or denial of service by some health workers, but not all. However, in our opinion, it is not acceptable to deny services to a pregnant woman because her partner does not turn up. As a result, if their partners do not attend ANC, this ultimately compromises birth preparedness. In a study completed in Kenya, women who feared HIV testing did not attend ANC (55). Moreover, it has been shown elsewhere that when women do not attend ANC, they will be less likely to deliver in a health facility (56). This contributes to ineffective birth preparedness and hence delay in seeking care for preventive care (57). Improvement in efforts to involve reluctant men, especially in the clinic set-up at ANC, is needed so that they are conducive to male participation. Furthermore, welcoming staff with good interpersonal skills, who provide education that caters to the needs of both partners, are needed.

Financial difficulties, distance to health facility, lack of preparation for transport, and quick onset of labour were hindrances to BP/CR and hence non-utilisation of facility delivery, a finding that is in agreement with other studies in low-income countries, including Tanzania (39, 58–62).

Whilst there is intention to deliver in hospital, sometimes it does not happen, and women deliver at home. Performing a behaviour is proximally determined by the intention to perform the behaviour (30). In this study, although there is a preference for facility delivery, some participants did deliver with the help of TBAs. A study completed in Bangladesh showed that the intention to use the health facility was a strong predictor for actual use of a health facility (57). Another explanation for the dissonance between planned and actual behaviour could be due to the lack of money for transportation, the availability of TBA, and an inability to recognise the progress of labour (56, 63). It is, therefore, important for health workers to provide proper information about the onset and progression of labour when providing birth preparedness messages.

BP/CR in the community entails saving money for buying supplies and for transportation. This may reflect the understanding of the community that the money will be needed in the case of referral for emergency complications. This finding has also been demonstrated in India, Uganda, and Ethiopia (17, 21, 64). The Tanzanian policy of maternal health services, including ANC and delivery services, states that these should be provided free of charge. However, it is not uncommon for health workers citing stock-outs to insist that women buy supplies. Families may nevertheless decide to buy food instead, which is more important in areas of poor socio-economic status. This will eventually lead to less use of facility delivery, as it was observed in Uganda and Tanzania that women may instead opt to use TBA services as they are cheap and readily available (44, 65).

Furthermore, the community members had to save money to comply with referrals provided by the health workers. This strategy involved sending the pregnant woman to stay nearer to the hospital or transferring the woman to stay with relatives who live in Dar es Salaam. This could be a costly undertaking and it has been shown to deter women from complying with referral advice (66). There might, therefore, be a need to establish maternity homes in communities that are far from the health facilities.

One of the components of BP/CR is the identification of blood donor in advance; however, it was not done in this study area. Instead, there was a misunderstanding that this service could be bought at the hospital. This is a misconception and is unethical because, as a policy, the selling of blood units is not allowed (67).

### Cues of action and self-efficacy

In spite of awareness of the benefit of deliveries at health facilities, the community members described challenges of arranging transportation to the health facilities, due to poverty. The HBM strategies that help to activate readiness are referred to as cues for action, and the community, especially the village government, is recommended to support families financially to act accordingly with their intentions and preferences, and to enable swift transportation in emergency situations. To increase self-confidence, the health providers need to recognise their important task to educate pregnant families in BP/CR, so they know how to act in situations related to childbirth.

Furthermore, the community still uses traditional medicine, especially during pregnancy and delivery. During pregnancy, herbs are used to ‘tie’ the pregnancy when a woman suffers repeated miscarriages. This practice has been in use for generations. The herbs used for labour are believed to prevent prolonged labour when delivery is at home. These are also used in hospital delivery for the same purpose. This has also been reported in studies done in developing countries such as Nigeria, Zambia, and Malawi (46, 51, 68, 69). Although the use of orthodox and unorthodox health services in pregnancy and delivery is not new, there is a need to address the issue that the use of these herbs could ultimately delay seeking care in the case of an emergency and, ultimately, a loss of self-efficacy. Availability of the traditional herbs may give a false impression that facility delivery is not important. The use of herbs in the community should be scientifically tested in research so as to identify those that are beneficial or rule out which are harmful.

### Trustworthiness

Four researchers, FA, AB, CKM, and EK, are fluent Swahili speakers and they have knowledge of the subject matter and qualitative research methods and are familiar with the study area. This, together with the use of analytical procedures involving our collaborators, ED and PA, both obstetricians with experiences of clinical and research work on the African continent, ensured credibility (36). The authors discussed differences that emerged in interpreting the findings and agreed on the final findings. Researchers from different backgrounds, that is, Tanzania and Sweden with their professions and pre-existing conceptions or beliefs, provided constructive discussions to ensure agreement on final categories and hence confirmability was observed. A detailed description of the study area, including context and selection criteria, was given so that readers could provide their opinion on the transferability of our findings.

### Strength and limitations

Many studies on BP/CR have been quantitative in nature and have reported calculated numbers of participants being ‘well prepared’. However, we have conducted a qualitative study, with the aim to explain the community's perception of how BP/CR is discussed and used, which we consider to be a strength in our study. We consider further the use of different participants in terms of age, and gender was strength because they provided different perspectives related to the topic of discussion. A possible limitation is that of the participants giving socially desirable answers rather than a genuine reflection. This could have occurred because the moderators and note-takers were of a medical background, and hence the participants may have had a desire to show preference for the use of skilled care. Another limitation is that the moderators were all male and this could have caused women to be shy and reserved. However, we believe this did not happen, as they were all vibrant and participated in dynamic discussions.

## Conclusions

This study shows that the perceptions of the community regarding BP/CR are in favour of skilled care. The community is aware of the importance of attending ANC, saving money for buying supplies, and understanding the importance of obstetric emergency care. However, issues related to distance, poverty, informal costs, inability to prepare in advance, and certain traditional beliefs and stigma towards unmarried women hinder the realisation of the intention to use skilled care. The community does not have a mechanism in place to help those who are in need in case of an emergency. It is important to innovate on how the community reinforces BP/CR, such as using insurance schemes, community health fund, and providing information on birth preparedness messages through among others community health workers. An additional option is to increase health promotion by developing a written and oral family programme on life-saving skills, including husbands, in-laws, and other persons significant to the pregnant woman, which could be delivered and kept in their homes.
